# The feelings of knowing – fundamental interoceptive patterns (FoK-FIP) system: connecting consciousness to physics

**DOI:** 10.1080/19420889.2023.2260682

**Published:** 2023-09-27

**Authors:** Holly Pollard-Wright

**Affiliations:** a Research, Institute of Electrical and Electronics Engineers (IEEE), New York, NY, USA; b Research, National Coalition of Independent Scholars, Brattleboro, VT, USA

**Keywords:** Consciousness, feelings of knowing (FoK), feelings of knowing - fundamental interoceptive patterns disorder (FoK-FIP D), fifth fundamental force, interoception dysfunction, mind

## Abstract

The feelings of knowing – fundamental interoceptive patterns (FoK-FIP) theory is both a theory of the mind and a unification theory. It includes cosmological and cellular frameworks. The cellular frameworks occur through the cosmological frameworks. This framework within a framework approach allows the connection between physics and consciousness to be envisioned in new ways, expanding current understanding and definitions. The cosmological frameworks refer to the astrophysics and theoretical physics constructs (e.g., string theory) that, without the use of mathematical language, conceptually expand the theory. In contrast, the cellular frameworks are the constructs represented by living organism models with DNA. In this way, the FoK-FIP theory represents an efficient framework for understanding consciousness and its phenomena. The transdisciplinary modeling of the FoK-FIP theory creates contextual bridging between classical theory and quantum theory as well as a broad range of empirical research so that biology and information connect, creating new avenues for disease diagnosis, intervention, and prevention. This article intends to render the FoK-FIP theory more robust and accessible for practical application by introducing the FoK-FIP system, which includes figures to promote clarity. Further, the theory aims to stimulate reasoning that challenges current notions about ‘life’ and the concept of ‘self.’ Through this process, the theory might contribute to the transdisciplinary collaboration needed to address some of the world’s complex issues. It is suggested that a significant contributor to the current complex matters in the world is the lack of understanding of how things are and how they appear.

## Introduction

This article aims to render the FoK-FIP theory that has been peer-reviewed and published [1] [2–4]; more robust and accessible for practical applications. Only by doing this can the theory contribute to solving the world’s complex issues. The FoK-FIP system represents the process in which ideas from the FoK-FIP theory, thoroughly discussed in the scientific literature, are removed from their intellectual content. Only those ideas that capture the dynamic of some generic process [5] are used to create the FoK-FIP system. This systematic approach facilitates a better understanding of the FoK-FIP theory, which is crucial to its practical relevance. The FoK-FIP theory includes cosmological and cellular frameworks in which the cellular occurs through the cosmological. This framework within a framework approach ensures that classical and quantum theories connect to consciousness in new ways that expand current understanding and definitions. The FoK-FIP theory combines biology and information theory with empirical research findings from various scientific disciplines. Transdisciplinary modeling is a core feature of the FoK-FIP theory that has the potential to reframe broadly scientific discussions that include physics and its laws and how those laws evolve. For example, in the FoK-FIP theory, the universe has five fundamental forces: electromagnetic, strong, weak, gravitational, and cognitive force. Further, the transdisciplinary modeling of the FoK-FIP theory creates contextual bridging needed to fill gaps in understanding related to biology so that medicine evolves using new physics to create avenues for disease diagnosis, intervention, and prevention. For example, the feelings of knowing – fundamental interoceptive patterns disorder (FoK-FIP D) is the maladaptive schema of the FoK-FIP theory connected to the cognitive force. In contrast, the feelings of knowing – fundamental interoceptive patterns mindfulness-based proprioception intervention (FoK-FIP MBPI) is the treatment representing new ways of thinking about epigenetics. Additionally, aspects of the FoK-FIP theory are being tested through an Institutional Animal Care and Use Committee approved pilot study (PLAVS IACUC Number: C001). This study using dog models with anxiety conditions is in its early stages; thus, this article will not make conclusions. It represents novel research based on the FoK-FIP theory that fills knowledge gaps in the literature regarding interoception dysfunction and emotional dysregulation correlated to a wide range of human and non-human animal models. This study’s aspects are mentioned briefly in this article to show how the transdisciplinary modeling of the FoK-FIP theory connects to empirical research. Further, the FoK-FIP system introduced in this article contains figures thematically composed to promote clarity and illustrate causal processes outlined by the FoK-FIP theory.

## Theory methodology

The FoK-FIP theory was developed around the central principle that transdisciplinary modeling must connect consciousness to new physics and contribute to scientific discovery through the following:

- The theory must aim to answer why phenomena occur and behave the way they do [6]. As such, thought experiment processes must fill gaps in the scientific literature by combining a broad range of experience and scientific knowledge base in creative ways to produce transdisciplinary modeling.
- Concepts developed by correlating a broad range of peer-reviewed literature must create new ways of thinking about reality to facilitate novel avenues for scientific exploration. However, expanding scientific definitions connected to concepts must be done in a way accessible to a wide range of audiences to facilitate transdisciplinary collaboration. As such, text and figures rather than mathematical language have been used to introduce the new physics of the FoK-FIP theory into the scientific literature. Nevertheless, the FoK-FIP theory is based on logic conceptually elaborated figuratively and thus can be further developed mathematically in the future.
- The theory and modeling must significantly contribute to animal (both human and non-human) welfare. This process is in the beginning stages and correlates with ongoing research. The transdisciplinary modeling of the FoK-FIP theory combines the new physics with the new disease FoK-FIP D and intervention FoK-FIP MBPI. Transdisciplinary modeled, the interoception of FoK-FIP dysfunction of the cognitive force correlates with mental health conditions in animal models (human and non-human). In the human model, interoception (i.e., the feeling of knowing what is happening in the body) is increasingly recognized as an important component of different mental health conditions, including anxiety disorders, mood disorders, eating disorders, addictive disorders, and somatic symptom disorders [7].

Anxiety-related disorders in human models are associated with a high burden of illness yet are often underrecognized and undertreated in primary care [8–10]. Further, whether non-human animals have emotions was historically a long-lasting question [11], with few researchers currently disputing that they do [12,13]. Separation anxiety is an anxiety-related disorder common in dogs and is observed in the owner’s real or perceived absence. Although this condition has been the most commonly discussed canine anxiety disorder in the literature, etiology, treatment, and prevention remain elusive [14]. The FoK-FIP theory correlates separation anxiety with a type of FoK-FIP D. The ongoing pilot study using dogs with anxiety conditions includes daily intervention consisting of the FoK-FIP MBPI. Observations in the testing environment are combined with infrared thermal imaging (IRT) to create baseline and post-intervention thermograms and the owner’s daily subjective assessment of the change in their dog’s behavior. This research shows how aspects of the FoK-FIP theory are currently being empirically tested.

## Core propositions

- The universe is non-separable through the mind.
- How things are and how they appear differ. Phenomena do not solidly exist as they seem to.
- Time and the distinction between ‘self’ and others are stubbornly persistent illusions.
- The key to solving the phenomenal world’s complex problems is to understand that behind the reactions of every animal model are the reactions of the cognitive force to the interoception of FoK-FIP (e.g., FoK-FIP interference or FoK-FIP feeling tones): automatic reactions – impulsive reactions – reactions with forethought may follow impulsive reactions

## An overview of the FoK-FIP theory

The FoK-FIP theory seeks to unify researchers through the peer-reviewed literature by enlarging the present scientific paradigm and going beyond the orthodox materialistic worldview. For example, by having first-person approaches in an otherwise too exclusively third-person-based science. Further, the FoK-FIP theory embraces the importance of the meaning of ‘life.’ It does this through transdisciplinary modeling that fills gaps in the scientific literature while aiming to make complex concepts broadly accessible to a wide range of audiences. The FoK-FIP theory includes an electromagnetic field theory of consciousness. It differs from other electromagnetic field theories of consciousness [15–17] partly because it is a theory of the mind and a unification theory that includes aspects of string theory. Additionally, the theory shows how physics and consciousness are connected through the relationship between two frameworks (i.e., the cosmological and cellular). This connection is essential because it aligns with the goal of theoretical physics and, specifically, string theory, to unify all interactions in ways that can be shown through empirical research. In the FoK-FIP theory, it is not strings themselves that unify; instead, it is the cognitive force whose role it is to unify the attributes of the mind interacting. This point is vital because what has been missing in the theoretical physics literature is the fact that unifying interactions through mathematical formulas is an act of cognition. It is an act that requires a high degree of inferential reasoning that needs a perceiver, which the FoK-FIP theory defines as the cognitive force. Furthermore, the FoK-FIP theory has shown that string theory is a unification theory, and the idea that it needs to be defined only through mathematical language is misleading. This arguably has contributed to string theory’s irrelevance. The fact that the FoK-FIP theory that uses aspects of string theory has led to experimental design producing empirical data related to tackling what may be one of the phenomenal world’s most pressing problems, anxiety conditions, shows its relevance. The ongoing IACUC-approved pilot study in canine models based on this theory has produced promising results related to the intervention (i.e., the FoK-FIP MBPI). The pilot study findings will be validated in the testing environment with the more extensive IACUC-approved study. Importantly, this ongoing pilot study has led to an experimental design in which the FoK-FIP MBPI is used to treat anxiety conditions in the human model introduced into the peer-reviewed literature [18]. This research in animal models (both human and non-human) aligns with the ‘holy grail’ of physics, redefined by the FoK-FIP theory in ways that correlate with the theory’s methodology clearly stated in this manuscript. The FoK-FIP theory is unique in that its transdisciplinary modeling of the cognitive force has filled gaps in understanding in the scientific literature in ways that have not been done previously. For example, the transdisciplinary modeling of cognitive broadcasting includes gravity, electromagnetism, and binding. Through cognitive broadcasting, the cognitive force unifies gravity with electromagnetism.

This aligns with the ideas of physicist Albert Einstein, who showed that the gravitational field equation is a symmetric mathematical structure. However, the FoK-FIP theory’s transdisciplinary modeling expands the understanding derived from Einstein’s theory to show (without using mathematical language) that gravity and electromagnetism are two sides of the same coin through perception, specifically the perception of the cognitive force. Aspects that distinguish the FoK-FIP theory in the scientific literature include the fact that the nonphysical and physical are always a part of transdisciplinary modeling. The term new physics refers to this aspect of the theory’s transdisciplinary modeling. An example of the theory’s new physics is the nonphysical awareness charge FoK. The core concept about the nonphysical awareness charge FoK related to physics is that it mimics the physical electric charge and leads to a nonphysical awareness current. This nonphysical awareness current, in turn, mimics the physical electric current occurring through the frameworks. It produces the nonphysical extremely low-frequency (ELF) magnetic field FIP. Further, FoK causes the emergence of the nonphysical cognitive force. Cognitive broadcasting includes the cognitive force binding to the electromagnetic field through a wavefront. The wavefront is produced through the coupling between the nonphysical and physical electromagnetic fields. The process of cognitive broadcasting includes FIP pushing the cognitive force. In order to bind, the cognitive force must be pushed by FIP against gravity in a process related to theoretical physics and biological processes through the theory’s framework within a framework approach (i.e., the cellular framework occurs within the cosmological framework). As such, this transdisciplinary modeling addresses both the binding problem in cognitive science and the unification of the forces of nature, a problem related to theoretical physics. The theory includes the components map model with interoceptive markers (IMs) to help figuratively envision the cognitive broadcasting process. Importantly, through FoK, there is a connection between the cognitive force (i.e., the nonphysical observer or perceiver of ‘life’) that cognitively broadcasts and the activity of the frameworks. The theory’s physics-derived mechanistic modeling of awareness charge in which awareness current follows explains where the cognitive force and ELF magnetic field FIP come from. It expands what is known about charge through hypotheses that allow the motion of charge to include coupling between the nonphysical and physical and, in doing so, connects a broad range of scientific fields. In this way, the theory’s transdisciplinary modeling represents new physics in which a crucial criterion is that the nonphysical and physical must be melded. The theory suggests its approach is crucial to how physics laws evolve, allowing theoretical physics (e.g., string theory) to be relevant and shown through empirical research in the testing environment. Physics and consciousness concepts always connect through the theory’s transdisciplinary modeling, including in modeling the universe. In the theory, the universe is non-separable and dynamic through the co-existence of energy, fields, matter, and consciousness.

Throughout this manuscript, the FoK-FIP theory’s transdisciplinary modeling includes using physical and nonphysical terms for clarification purposes. When the term physical is used, it refers to phenomena that can be directly experimentally measured in the phenomenal world. Physical refers to physical theory (i.e., one or more relationships between measurable quantities). In contrast, when nonphysical is used, it refers to the theoretical part of transdisciplinary modeling derived from knowledge about phenomena that cannot be directly experimentally measured but can be indirectly measured and correlate with aspects of consciousness. The theory’s internal consistency includes the cognitive force that unifies interactions through perception, which speaks to the FoK-FIP theory’s core propositions: how things are and how they appear differ. Phenomena do not solidly exist as they seem to. The theory’s transdisciplinary modeling updates the concept of the cognitive force of the universe described in the literature through the ages. It does so in ways that allow the concept to fill in knowledge gaps in the scientific literature connected to empirical research. For example, when broadcasting signals cognitively, the cognitive force combines distinct parts of the electromagnetic field in a process related to perception, decision, and action. A core concept related to cognitive broadcasting is that nonphysical and physical are terms related to the phenomenal world of interoceptive cognition consciousness produced by the cognitive force that broadcasts signals cognitively.

## The FoK-FIP system’s core components with fundamental concepts

- **The mind** is the entity in the FoK-FIP theory without a beginning or ending and with neither increase nor decline [19]. It is the source of everything. Transdisciplinary modeling, therefore, answers the big question of where everything came from in a way that prevents another similar question and a fruitless feedback loop (e.g., “So where did that come from, and where did that come from, and where did that come from?” and so on). Further, the mind has infinite potential; when its potential manifests, the mind’s attributes emerge. The mind itself does not change when this happens. That abstract idea correlates the mind to the law of conservation of energy (without mathematical language). This law states that a fundamental quantity called energy does not change in the manifold changes that nature undergoes. It is an abstract idea because it is a mathematical principle that says there is a numerical quantity that does not change when something happens (The Feynman Lectures on Physics, 1970) [20]. This principle is not a description of a mechanism or anything concrete, but a strange fact that some number can be calculated, and “when we finish watching nature go through her tricks and calculate the number again, it is the same.” - Physicist Richard Feynman. Further, in the FoK-FIP theory, change and symmetry refer to the mind’s attributes, not the mind itself. This is also an abstract idea related to the mind and refers to why the mind itself cannot be fully realized. The concept here is that some aspects of the mind cannot be known. This is because change does not apply to the mind itself. Something can be fully realized only with change, and specifically, change with a pattern.
- **Change** occurs through the mind manifesting its infinite potential in which attributes of the mind emerge. It refers to the attribute itself (e.g., dark energy, focal points of dark matter, normal matter, and cognitive force). Change can be patternless or patterned based on the variation associated with the state (e.g., pure awareness, pure mental, mental images, or ‘distress’) of the attribute that cannot be or can be directly or indirectly empirically measured. For example, focal points of dark matter (FPDMs), normal matter, and cognitive force refer to patterned change, whereas dark energy is patternless.
- **Symmetry** refers to what the attribute with the state produces that cannot be or can be directly or indirectly empirically measured. In the theory, it is associated with both transformation and the universe’s fundamental forces.
- **Transformation** refers to metamorphosis using an expanded definition. It occurs through the mind’s attributes and is either induced through interaction or occurs spontaneously. It always includes change and can lead to a dramatic symmetry in form or appearance.
- **Awareness** is a concept that the theory has expanded by associating it with dark energy. This concept has aspects that align with astrophysics’ hypothesis that dark energy is associated with a constant energy density, possibly the point-zero energy of fields. In the FoK-FIP theory, the universe’s substrate is dark energy with the pure awareness state in which interactions between the attributes of the mind occur. Through cosmological frameworks in which the universe’s substrate is dark energy, and in a general sense, pure awareness is a state that can be construed as underlying that which connects the pure mental state (of FPDMs), the mental image state (of normal matter), and the ‘distress’ state (of the cognitive force). This idea then connects with how the theory has modeled the non-separable universe with consciousness.
- **‘Consciousness’** has an expanded definition in the theory. The theory’s transdisciplinary modeling includes that ‘Consciousness’ refers to the potential for interactions between the attributes of the mind, specifically dark energy with the pure awareness state and normal energy with the mental image state. In this way, ‘Consciousness’ refers to the pre-information stage related to the interaction between the attributes of the mind.
- **The universe** described in the literature [21], consisting of approximately 68% dark energy, 27% dark matter, and 5% normal matter, are attributes of the mind. Further, the universe has five fundamental forces that occur through attributes of the mind interacting: electromagnetic, strong, weak, gravitational, and cognitive force. Attributes of the mind have states: dark energy with the pure awareness state, dark matter referred to in theory as focal points of dark matter (FPDMs) with the pure mental state, and normal matter (also referred to in the literature as matter or ordinary matter) with the state of mental images and the cognitive force with the state of ‘distress.’
- **Electromagnetic radiation (EMR) consciousness**, in the theory, refers to the information produced when the attributes of the mind interact. There are two forms of EMR consciousness: generalized and localized.
- **Generalized electromagnetic radiation (EMR) consciousness (i.e., Universal Consciousness)** in the theory occurs through the attributes in the mind interacting and refers to universal consciousness. In a generalized way, it occurs in the universe and within cosmological frameworks.
  **Generalized EMR consciousness** = nonphysical EMR + physical EMR + the pure awareness state of the universe’s substrate dark energy.
- **Focal points of dark matter (FPDMs)** Dirichlet membranes (i.e., D-branes) are an important class of branes in string theory that the FoK-FIP theory re-envisions through transdisciplinary modeling of FPDMs. D-branes of FPDMs occur when parts of the universe’s dark energy substrate transform. The configuration of a single focal point of dark matter (FPDM) consists of a focal point of multi-dimensional space surrounded by an inner and outer layer of open and closed dark energy strings. The focal point of multi-dimensional space is the source of gravity, whereas the inner and outer string layers are the source of entropy. This modeling includes that open and closed strings propagate through spacetime because their endpoints lie on a focal point of multi-dimensional space. Through cosmological frameworks, the inner and outer string layers of FPDMs can do two important things related to charge and the behavior of the cognitive force: (a) produce nonphysical magnetic charge and (b) nonphysical awareness charge FoK. This dual aspect of the strings of FPDMs, specifically the awareness charge, is transdisciplinary modeled as related to transforming the universe’s dark energy substrate with pure awareness. Further, through cosmological frameworks, FPDMs are the ‘tiny’ black holes with hot bodies within the framework that produce the theoretical thermal black-body radiation associated with localized electromagnetic radiation (EMR) consciousness. This concept has aspects that align with Hawking radiation (i.e., describes hypothetical particles formed by a black hole’s boundaries).
- **Cosmological frameworks** The transdisciplinary modeling of the FoK-FIP theory for cosmological frameworks includes that FPDMs are the source of magnetic monopoles. This means they can produce magnetic charge and magnetic current. In contrast, when normal energy converts to normal matter (in events related to the universe’s formation), it becomes the source of electric monopoles. This means normal matter can produce electric charge and electric current. The core idea here is that through cosmological frameworks, FPDMs have simultaneous direct relationships with the (a) universe’s dark energy substrate with the pure awareness state, (b) normal matter with the state of mental images that embodies it, and (c) the cognitive force with the ‘distress’ state that emerges from it. In contrast, through the cosmological frameworks, normal matter has only a direct relationship with FPDMs. It indirectly interacts with the universe’s dark energy substrate and cognitive force. Further in the universe, FPDMs are the entities that, through cosmological frameworks, produce nonphysical aspects of ‘life’ (i.e., interoceptive cognition consciousness). In contrast, normal matter is the entity from which the physical aspects of ‘life’ come. These relationships are integral to creating the conditions that allow the cognitive force to emerge within frameworks and cognitively broadcast to produce interoceptive cognition consciousness (i.e., ‘life’) with a phenomenal world. FoK is the hidden variable, whereas FIP is the variable integral to how the cognitive force is connected to the activity occurring through the cosmological frameworks.
  **Through FPDMs**: nonphysical awareness charge FoK – nonphysical awareness current mimics the physical electric current produced by normal matter and melds with the nonphysical magnetic current produced by FPDMs – nonphysical ELF magnetic field FIP

**FIP**:

1. Pushes the cognitive force to bind to the wavefront (i.e., consisting of the nonphysical and physical electromagnetic field) of localized electromagnetic radiation (EMR) consciousness (generated by FPDMs).
2. Pulls the cognitive force toward the ‘tiny’ black hole (i.e., the focal point of multi-dimensional space of FPDMs). At the same time, localized EMR consciousness is configured into a conical shape (i.e., conical EMR consciousness) through gravity.
3. **Localized electromagnetic radiation (EMR) consciousness** is a specialized type of EMR consciousness produced through the configuration of FPDMs of cosmological frameworks and refers to individual consciousness. Further, related to the configuration of FPDMs, localized EMR consciousness configures into a conical shape. It irradiates the surface of a focal point of multi-dimensional space. Conical EMR consciousness is the FoK-FIP theory’s symbolic representation of the inverse square law of light and a unique space-filling field (without using mathematical language). It refers to an area within cosmological frameworks in which the electromagnetic field exists in a way that increases as the square of the distance from the FPDMs’ focal point of multi-dimensional space surrounded by inner and outer string layers. Additionally, through FPDMs, the pure mental state emits from the ‘tiny’ black hole akin to light brightness. In this process, the pure mental state of FPDMs decreases with the inverse square of the distance. Through the relationship between FPDMs and the cognitive force of cosmological frameworks, localized EMR consciousness is data with signals derived from the electromagnetic field that will be cognitively broadcast. Integral to this process is FIP, and that localized EMR consciousness has a wavefront.
  **Localized EMR consciousness (i.e., individual consciousness)** = nonphysical EMR + physical EMR + FPDMs with the pure mental state embodied by normal matter with the state of mental images
4. **Wavefront** is where the cognitive force cognitively broadcasts signals. The wavefront occurs through the coupling between the nonphysical and physical electromagnetic fields of localized EMR consciousness. The wavefront is important to developing the relationship between FPDMs and the cognitive force explaining complex perception aspects. A significant contributor to this complexity is that cognitive broadcasting by the cognitive force causes the cellular frameworks to emerge within the cosmological frameworks.
  **The wavefront of localized EMR consciousness** = nonphysical electromagnetic field + physical electromagnetic field
5. **Feelings of knowing (FoK)** [22] is a term the theory repurposes to mean a nonphysical charge occurring through frameworks (e.g., cosmological and cellular). It is a transdisciplinary construct because it includes the awareness concept of cognitive science and the charge concept of physics. FoK causes both the emergence of the cognitive force and produces nonphysical awareness current. It explains how the cognitive force can be correlated with the theory’s framework within a framework approach. Further, FoK is construed in the theory as the universe’s *hidden variable* through its relationship to the cognitive force of the frameworks. In the cosmological frameworks, FoK begins through some closed strings on the inner string layer of FPDMs and propagates to the outer string layer, moving radially through the awareness current. In contrast, in the cellular frameworks, through FoK-gene expression, FoK occurs. Awareness current propagates through the activity of the cells of the cellular frameworks with DNA.
6. **Cognitive force** is the hypothetical nonphysical force. It is both an attribute of the mind, specifically a synthesizing attribute, and the fifth fundamental force of the universe. The idea of a “universe with a cognitive force” is as old as humanity. It can be found throughout several cultures and in all times, beginning from the indigenous cultures, through Spinoza, the German idealists, and many Eastern philosophies (which are nowadays going through a revival in modern philosophy with panpsychism, cosmopsychism, and analytic idealism). Nevertheless, there is a distinction between how the theory contextualizes the cognitive force. The theory interprets the cognitive force using technical words and philosophical knowledge, combining the concept with modern medical knowledge, health research, and contemporary physics. By doing that, the FoK-FIP theory may provide another new dimension of knowledge of metaphysics related to the cognitive force. Further, the cognitive force represents new physics because it occurs with the physical electromagnetic force after emerging from FoK.
7. **Electromagnetic force (EMF)** is a physical force. In the FoK-FIP theory, EMF has an expanded definition. EMF is the force of interactions between electrons and a magnetic or electric field that always occurs with cognitive force.
8. **Macrocosm** has an expanded definition in the theory. It refers to the place in the universe outside cosmological frameworks.
9. **Microcosm** has an expanded definition in the theory. It refers to the place within cosmological frameworks linked to the macrocosm through the universe’s substrate dark energy with the pure awareness state.

### The core component of the FoK-FIP theory associated with cognitive broadcasting

- **Cognitive broadcasting** is a process in which the cognitive force of a cosmological framework cognitively broadcasts the signals generated through interactions between the attributes of the mind (i.e., dark energy, normal matter, and FPDMs). There are many cosmological frameworks in the universe and, thus, many viewpoints of the cognitive force that broadcasts signals cognitively.
  **Cognitive broadcasting** = localized EMR consciousness + cognitive force + wavefront + gravity + FIP
- **Viewpoint = Cognitive force perceiving cognitive broadcasting.** Viewpoint in this context refers to the process where the cognitive force reacts to the interoception of FoK-FIP (e.g., FoK-FIP interference and FoK-FIP feeling tones) with varying degrees of sensitivity. By doing that, it makes decisions, allowing it to perceive signals broadcast cognitively.
- **Fundamental interoceptive patterns (FIP)** is a term the theory uses to mean the nonphysical extremely low-frequency (ELF) magnetic field produced through nonphysical awareness current. FIP is integral to the cognitive broadcasting process.
- **The interoception of FoK-FIP** is the substrate for cognitive broadcasting and is a term that refers to both FoK-FIP interference and FoK-FIP feeling tones.
- **FoK-FIP interference** refers to the process where nonphysical awareness charge FoK interferes with the nonphysical ELF magnetic field FIP. Important to this process is the awareness current that produces FIP. Further, the cognitive force reacts to FoK-FIP interference but is unaware it is doing this.
- **FoK-FIP feeling tones (e.g., pleasant, unpleasant, or neutral)** are the immediate and spontaneous awareness of the cognitive force with varying degrees of sensitivity to the frequency of FoK and intensity of FIP. FoK-FIP feeling tones are important to how the cognitive force interprets its relationship with FPDMs of cosmological frameworks with an awareness that may include feeling heat and/or unpleasant, pleasant, or neutral patterns of FIP.
- **Interoceptive cognition consciousness** is ‘life’ according to the FoK-FIP theory. The cognitive force cognitively broadcasts signals generated through the cosmological frameworks. This produces interoceptive cognition consciousness. In this process, signals broadcast cognitively create versions of a three-dimensional phenomenal world. This world has living organism models with DNA, places, and things. Further, the cognitive force may understand the phenomenal world as having four fundamental forces of nature: electromagnetic, strong, weak, and gravitational. In this way, the cognitive force that cannot perceive its relationship to FPDMs nor ‘see’ itself directly can model a version of its ‘life.’ This ‘life’ ultimately is the life of attributes of the mind interacting through cosmological frameworks. The theory’s transdisciplinary modeling of cognitive broadcasting by the cognitive force that produces interoceptive cognition consciousness has aspects similar to the Cit-Shakti of tantrism, the ‘conscious energy,’ and Spinoza, in which matter and mind are ‘modes of existence of one and the same substance.’
- **Living organism models with DNA** are electromagnetic figures representing cellular frameworks. They emerge within the phenomenal world of interoceptive cognition consciousness when the cognitive force is broadcasting for higher-end cognition. The distinctive way signals are cognitively broadcast by the cognitive force based on its sensitivity to the interoception of FoK-FIP is represented by living organism models with DNA. These models may appear to the cognitive force as solid. As such, they represent how interoceptive cognition consciousness can be misleading. This concept aligns with ideas derived from an electromagnetic paradigm for biology and medicine theory (see [23]).
- **The components map model with interoceptive markers (IMs)** Cognitive broadcasting can be envisioned through the components map model with interoceptive markers (IMs). The model facilitates understanding of what happens when the cognitive force broadcasts signals. It is a process that correlates to the state-dependent dynamics of quantum mechanics. Without using mathematical language, cognitive broadcasting correlates with a well-understood mathematical prescription known as unitary evolution. Regarding unitary evolution, a pure quantum state is described by a coherent wavefront that, left in isolation, will evolve predictably. However, when a measurement is made, the state changes abruptly. This phenomenon is often called the collapse of the wave function. In this process, the state change projects the system into one possible eigenstate (i.e., a state of a quantized dynamic system) corresponding to the observable being measured. For that ideal measurement to occur, the unitary evolution rule is replaced by the Born rule, which predicts the relative probabilities of the measurement outcomes. This process introduces the element of indeterminism or uncertainty into quantum mechanics and the transition from the quantum to the classical domain. The FoK-FIP theory includes the cognitive force that cognitively broadcasts, with formal theoretical cellular and cosmological frameworks, connects information to matter. The theory deepens understanding of the informal dictum, Life = Matter + Information [24].

### The highlights of the FoK-FIP theory’s transdisciplinary modeling of the universe with consciousness

- The mind – The mind’s infinite potential manifests – Attributes of the mind emerge – The universe manifests (eventually) correlated with attributes of the mind interacting

According to the FoK-FIP theory, the mind existed (and still exists) before the Big Bang event, which refers to the physical theory that describes how the universe expanded from an initial state of high density and temperature. The FoK-FIP theory’s transdisciplinary modeling includes the idea that after the mind manifests its infinite potential (before the universe’s formation), dynamical energy (or a field), dark energy with the pure awareness state, and normal energy with the mental image state occurs. Additionally, in the FoK-FIP theory, there is more dark energy after the mind manifests its potential than normal energy. This part of the theory’s transdisciplinary modeling was derived from contemporary astrophysics. The literature reports that the universe has more dark energy than normal matter (in the theory, normal energy transforms into normal matter). Because of this difference, in the FoK-FIP theory, these attributes cannot interact. Therefore, dark and normal energy transformation was needed to facilitate interaction. Furthermore, dark and normal energy have states (i.e., pure awareness and mental images, respectively) and, thus, the ingredients needed for consciousness to exist. According to the FoK-FIP theory, the mind’s emerging attributes tend to interact, and by doing this, a universe manifests in stages that correlate with the terms macrocosm and microcosm. The concept that the mind’s attributes tend to interact when they emerge is a foundational schema of the theory. This concept has aspects that correlate with the anthropic principle, also known as the “observation selection effect,” a hypothesis first introduced into the literature by astronomer and physicist Robert Dicke, but there are differences. In the FoK-FIP theory, the mind does not, in a predetermined way, decide how its attributes interact so that something is created. Instead, the fact that the mind’s attributes interact when situated together is based on a trend (i.e., when together, the mind’s attributes tend to interact). In this way, the theory’s transdisciplinary modeling explains why the transformation between the mind’s attributes occurs. These transformations include the one in which energy converts to matter only after the universe expands and cools. This modeling without mathematical language has aspects that align with physicist Albert Einstein’s formula E = mc2, in which energy and mass (i.e., matter) are interchangeable. This means that under the right conditions, energy can become mass and vice versa. In the FoK-FIP theory, the universe with consciousness evolves in stages that begin with the transformation of dark energy into FPDMs, in which normal energy’s transformation into normal matter follows. This modeling of the FoK-FIP theory aligns with aspects of the Big Bang Theory in which the universe was so hot after this event that matter formation was prevented from occurring. It also aligns with the quantum vacuum, a hypothesis explaining how the universe came into existence in a vacuum that contained no matter.

The FoK-FIP theory’s transdisciplinary modeling includes the concept that parts of the universe’s dark energy transform, which correlates with the emergence of FPDMs. After emerging, FPDMs receive a ‘cosmic jump-start’ from dark energy consisting of pure awareness. In this process, on the inner layer of FPDMs, some closed strings convert from the ground state to the excited state. This event begins through some closed strings on the inner string layer and propagates to the outer string layer, moving radially. The core idea here is that the ground state of strings in the macrocosm is derived from the pure awareness state of the universe’s dark energy substrate. In contrast, the rate of string conversion from the ground state to the excited state is directly proportional to the concentration of the pure mental state of FPDMs. Gravity is produced through FPDMs, specifically the focal point of multi-dimensional space with vibrating strings. Gravity then pulls normal energy to FPDMs; in this process, normal energy converts to normal matter. At the same time, it enfolds FPDMs to correlate with the idea that normal matter contains or cosmically embodies FPDMs. Through FPDMs, the FoK-FIP theory incorporates string theory in ways that connect the theory to Einstein’s theory of General Relativity (currently the best working theory of gravity). Importantly, in the macrocosm, the cognitive force is only a potential attribute of the mind. It will manifest through its relationship to FPDMs and emerge in the microcosm within the cosmological frameworks. This concept includes the idea that signal processing becomes more complex along with the stages of the universe’s history and correlates with cognitive broadcasting by the cognitive force. In the FoK-FIP theory, the role of the cognitive force is to synthesize relationships between the attributes of the mind interacting through cosmological frameworks. Integral to this process is the idea that dark energy with pure awareness that creates the universe’s landscape must transform. The core relationship related to the cognitive force comes through FPDMs of cosmological frameworks. In this way, the cognitive force is a construct that fills what was alluded to most notably by the architects of quantum mechanics – Niels Bohr, Eugene Wigner, and Werner Heisenberg – who felt there is something new and different in the physics of living matter [24].

### The Einstein-Podolsky-Rosen (EPR) argument in quantum theory expanded by the FoK-FIP theory

The statistical quantum theory based on physicist Niels Bohr’s ideas is developed in the FoK-FIP theory without mathematical language but rather transdisciplinary modeled activity occurring through the cosmological frameworks. The FoK-FIP theory expands the classical theory of black holes by configuring FPDMs of cosmological frameworks that incorporate concepts related to quantum mechanics and gravity. The cosmological frameworks in the microcosm represent quantum systems connected by the pure awareness state of the universe’s substrate dark energy. The theory’s transdisciplinary modeling of this relationship has aspects that align with the Einstein-Podolsky-Rosen (EPR) Argument in Quantum Theory. The EPR argument discussed how two quantum systems interact in such a way as to link both their spatial coordinates and their linear momenta in a certain direction, even when the systems are widely separated in space. In the FoK-FIP theory, the dark energy substrate facilitates information traveling between the cosmological frameworks at speeds that exceed the speed of light. It does this through the pure awareness state that represents change without a pattern. Dark energy with the pure awareness state acts as the universe’s hidden ‘information superhighway.’ Further, each FPDM has its own position on the universe’s dark energy substrate. Through this relationship, FPDMs receive change without a pattern from dark energy and convert it to change with a pattern through the vibrations of the inner and outer layers of strings. In this way, through the vibration of strings that encircle multi-dimensional space, the configurations of FPDMs with the pure mental state allow them to act as ‘cosmic computers.’ They can simultaneously process their relationship with dark energy, normal matter, and cognitive force. In contrast, the cognitive force cannot triple-process information at the speed of FPDMs. Instead, the cognitive force cognitively broadcasts the information it receives from FPDMs in a process that cannot exceed the speed of light. Further, in cognitive broadcasting, the cognitive force has no direct contact with normal matter or dark energy. Instead, the cognitive force is indirectly connected to these attributes through its relationship to FPDMs with dynamics that occur through the wavefront of the conical-shaped localized EMR consciousness. Through gravity derived from FPDMs that configure the conical structure of localized EMR consciousness, the wavefront consists of electromagnetic waves looped into an extremely fine fabric or network. This concept aligns with the approach of physicists Carlo Rovelli and Lee Smolin called loop quantum gravity [25].

In the FoK-FIP theory, the cognitive force through the wavefront cognitively broadcasts signals to create the three-dimensional world of interoceptive cognition consciousness. The cognitive force through higher-end cognitive broadcasting produces objects of interoceptive cognition consciousness. The theory’s transdisciplinary modeling of higher-end cognitive broadcasting aligns with physicist Leonard Susskind’s theory of the world as a hologram [26]. In this process, the cognitive force perceives many geometric pictures or images in the phenomenal world. Further, a ‘strange loop’ exists through the relationship between FPDMs and the cognitive force. This means that the vibrations of strings of FPDMs that produce FoK give rise to the observers of the cognitive force, who, in turn, conceive of the phenomenal world. Cognitive broadcasting through the wavefront of localized EMR consciousness includes the cognitive force acting as the ‘cognitive magnet.’ FIP is integral to the activity of the cognitive force because its role is to push and pull the cognitive force against and with gravity produced by FPDMs. The initial stages of cognitive broadcasting include the cognitive force radiating through the electromagnetic field of the wavefront before binding to create cognitive pixels. As such, the cognitive force is the anti-gravity force that correlates with the initial stages of cognitive broadcasting with binding. In this context, the term pixel does not match current definitions but expands the definition to include through the wavefront a unit of meaning and a physical unit. Through the process of cognitive broadcasting, the wavefront correlates to a vector field on a plane. It consists of coupling the nonphysical and physical electromagnetic fields in which cognitive pixels are dispersed throughout the wavefront. Therefore, the wavefront includes a collection of cognitive pixels with cognitive force. While the conical shape of localized EMR consciousness is formed, FIP pulls the cognitive force. The concept here is that FIP pushes the cognitive force through the wavefront fabric or network to bind, and it pulls so the cognitive force can be part of the conical structure of localized EMR consciousness. Through the localized EMR consciousness wavefront, cognitive pixels correlate to field vectors bound by the cognitive force to different space points at different time moments. This concept aligns with aspects of physicist David Bohm’s ideas about the relationship between mind and matter [27]. Further, cognitive broadcasting cycles include myriad cognitive pixels (e.g., many billions) brought together by the cognitive force simultaneously, no matter how far apart they are in the wavefront. In the FoK-FIP theory, cognitive broadcasting cycles through the localized EMR consciousness wavefront. A cycle of cognitive broadcasting correlates with cognitive cycles discussed in the literature [28]. It ends with the conical EMR consciousness pulled into the ‘tiny’ black hole of FPDMs before it reforms again, in which gravity is integral to this process.

The cognitive force is the cognitive magnet with FoK-derived intrinsic and FIP-derived extrinsic polarity, allowing it to ‘pull itself together.’ This concept correlates to the chunking and stimulus processing discussed in the consciousness literature [29]. The theory’s transdisciplinary modeling includes the idea that the cognitive force has a limit to how many cognitive pixels it can cognitively broadcast. In this process, FoK-FIP interference is the substrate for the massive parallelism aspect of cognitive broadcasting. This concept has aspects that align with a cognitive theory of consciousness [30]. In contrast, FoK-FIP feeling tones are the substrate for the flow of isolated conscious items corresponding to higher-end cognitive broadcasting. The wavefront of localized EMR consciousness, where cognitive broadcasting occurs and allows the cognitive force to synthesize information, has aspects that align with the global workspace theory (i.e., a theory of human cognitive architecture and consciousness) of neuroscientist Bernard Baars. The critical role of FPDMs related to cognitive broadcasting is to produce the *hidden variable* FoK from some closed strings on the inner layer. FoK-FIP interference, in which the cognitive force reacts automatically, represents a large part of cognitive broadcasting over which it has little control. In contrast, impulsive reactions to FoK-FIP feeling tones afford the cognitive force some control of the process; when initiating a reaction with forethought, the cognitive force has the most control, which correlates with ‘self-efficacy.’ Through degrees of inferential reasoning that emerge through higher-end cognitive broadcasting, the cognitive force will believe certain things about the phenomenal world it creates. In this process, it will understand aspects of ‘life.’ Its belief in what is ‘real’ or ‘not real’ often hinges on what it can perceive directly in the phenomenal world. The core concept here is that the cognitive force can neither perceive itself nor any parts of the cosmological framework that explain how it exists (e.g., FPDMs and the closed strings that produce FoK). In the phenomenal world, magnetism is understood to be somewhat analogous to electricity and is shown by a magnetic field with a direction defined as running from north to south. Importantly, there is no observed magnetic counterpart in the phenomenal world of electric charge. Instead, the magnetism is consistent with physicist James Maxwell’s equations, which describe the unification of electric and magnetic field theory into classical electromagnetism. This means the magnetism observed in daily ‘life’ can only be attributed to the movement of electric charges. In the FoK-FIP theory, what underlies daily ‘life’ is a hidden world that occurs through cosmological frameworks. It is a cosmic world where the nonphysical and physical coexist in non-separable ways. Through this transdisciplinary modeling, the FoK-FIP theory explains (without using mathematical language) why the elusive magnetic monopole predicted by quantum physics has not been seen in the phenomenal world.

### The overview of the FoK-FIP theory’s use of aspects of string theory

The theory’s transdisciplinary modeling (done without mathematical language) shows that through the configuration of FPDMs, there are quantum mechanical effects that expand classical theory. In classical theory, black holes can absorb but not emit particles, whereas, through the cosmological frameworks, FPDMs both absorb and emit particles in a process associated with localized EMR consciousness. The focal point of multi-dimensional space as part of the configuration of FPDMs is the ‘tiny’ black hole with surface gravity. It refers to the hot part of the ‘body’ of a single FPDM that creates and emits particles through the vibrations of the inner and outer string layers. In the FoK-FIP theory, string conversion occurs through the cyclic, although not repetitive, relationship between a FPDM and the cognitive force. FPDMs emerge from the universe’s dark energy substrate with equal open and closed strings. The number of strings does not change, but strings can convert (open strings convert to closed strings, and closed strings convert to open strings). String conversion occurs through FPDMs responding to the volitional reactions of the cognitive force (i.e., an impulsive reaction only or an impulsive reaction followed by a reaction with forethought). The core idea is that the cognitive force affects FPDMs with two types of strings: open and closed. String conversion describes the standard model of their relationship, representing theoretical perspectives of string theory. In this light, the theory’s transdisciplinary modeling is connected to the Higgs boson because they both support the idea that string theory is a justified approach to nature. In the FoK-FIP theory, the cognitive force is required for the phenomenal world to have a property called supersymmetry. Importantly, a supersymmetric Standard Model with string theory boundary conditions has Higgs bosons and explains their properties because of the fact that unifying interactions through mathematical formulas are an act of cognition. An explanation of anything requires a degree of inferential reasoning related to a perceiver that the FoK-FIP theory defines as the cognitive force. Further, over the course of many interactions through cosmological frameworks, thermal emission associated with localized conical EMR consciousness (that gets pulled into the ‘tiny’ black hole) of FPDMs leads to a slow decrease in the mass of the focal point of multi-dimensional space. This means that eventually, through their relationship, the volitional reactions of the cognitive force cause the disappearance of the FPDMs. Although these quantum effects violate the classical law that the area of the event horizon of a black hole cannot decrease, there remains a Generalized Second Law. The theory’s transdisciplinary modeling includes this second law connected to the’ Big Transformation’ event in the discussion section of this manuscript. This event represents where the stings of FPDMs transform back into the universe’s dark energy substrate. This means that the inner and outer string layers disappear into the pure awareness state of dark energy. Additionally, this transdisciplinary modeling shows (without mathematical language) that gravitational collapse converts baryons and leptons (produced through string vibrations) in the collapsing body of FPDMs into entropy. It explains why the universe contains so much entropy per baryon. This transdisciplinary modeling aligns with how Steven Hawking envisioned particle creation by black holes [31]. The highlights of the FoK-FIP theory’s use of aspects of string theory are as follows:

- The FPDMs’ inner string and outer string layers vibrate. These vibrations are linked to the mass of an elementary particle. The more a string vibrates, the more energy and, thus, the more massive it is.
- Endpoints are created through the strings’ orientation on the inner and outer layers of FPDMs. The strings are stuck on the focal point of multi-dimensional space. The strings carry charges and align with what is historically known as “Chan-Paton factors” at their endpoints.
- The endpoints behave as quarks. This means that if the string is oriented and carries an arrow from the ‘beginning’ to the ‘end,’ the beginning may be called a quark, and the end may be called an antiquark.
- Through the configuration of FPDMs, charges are analogous to point-like particles. Because of the inner layer of open and closed strings, the seemingly point-like origin of charges may be dual and equivalent to how strings produce the charges. Further, the pure mental state of FPDMs is how the strings know how to vibrate. As such, aspects of consciousness are part of how the mass of elementary particles is determined. In this process, the more the string vibrates, the more energy and the more massive the elementary particle.
- On the inner layer, the open and closed string is simply a string that moves in the opposite direction related to the curvature of the string. In contrast, on the outer layer, associated with the surface of multi-dimensional space, movement differs between open strings that attach and closed strings that “tap in.” On the outer layer, the FoK-FIP theory offers a unique, more intrinsically stringy origin of charges. Closed strings may wrap around in a non-contractible loop in spacetime related to the multi-dimensional space of FPDMs. This concept aligns with the circle from the Kaluza-Klein theory related to boundary conditions on the string. In this process, a particle with the opposite charge moves in the opposite direction along the hidden circular dimension of the configuration of FPDMs.
- Through cosmological frameworks, strings in the ground state act as ‘antennae’ of FPDMs that help them interact with normal matter with the mental image state. Further, strings change from the ground to excited states based on the FPDMs’ simultaneous relationship to normal matter and dark energy with the pure awareness state. Important to this process is the pure mental state of FPDMs.
- Through the relationship of FPDMs to the cognitive force, point-like particles in higher-dimensional spacetimes are linked to higher-end cognitive broadcasting. Further, the excited states of FPDMs’ vibrating strings correlate with the ‘distressed’ state of cognitive force related to FoK-FIP interference. In contrast, FoK-FIP feeling tones are how the cognitive force feels its relationship to FPDMs, which may include heat and/or patterns of FIP.
- The formation of localized EMR consciousness with a wavefront through cosmological frameworks is the quantization process that explains the relationship between FPDMs and the cognitive force. This relationship begins with the awareness charge FoK through some closed string (i.e., magnetic monopoles) on the inner layer of FPDMs. Important to the FoK-FIP theory’s transdisciplinary modeling where the nonphysical and physical connection occurs is the idea that where there is a charge (e.g., magnetic, awareness, or electric), a current (e.g., magnetic, awareness, or electric) follows. Through processes related to the wavefront, awareness (i.e., charge and current) *melds* with magnetic (i.e., charge and current) and *mimics* electric (i.e., charge and current). This modeling has aspects that align with the thinking of Nobel Laureate Paul Dirac, who showed that electric charge can exist only in discrete values through a ‘quantization’ process when Maxwell’s equations are extended to include a magnetic monopole. Electric charge is a requirement of quantum mechanics, and Dirac’s work showed that classical electromagnetism and quantum electrodynamics were compatible theories in this sense.

### The framework within a framework approach related to empirical research

The cognitive force exists in both the cosmological and cellular frameworks through the FoK-FIP theory’s transdisciplinary modeling with the framework within a framework approach. The cognitive force is bound to parts of the localized EMR consciousness wavefront that occurs through cosmological frameworks. In contrast, in the cellular frameworks, the cognitive force is part of the coupling between the physical and nonphysical electromagnetic fields associated with living organism models with DNA (representing the cellular frameworks). For example, this coupling is associated in animal models, such as humans or canines with a spinal cord and peripheral nervous tissue connecting the body and brain. In plant models, the stem is important to this coupling. The FoK-FIP theory has aspects that align with theories of plant consciousness [32]. The core concept about the FoK-FIP theory’s modeling of this physical and nonphysical coupling is that it takes place through the structure(s) that can be correlated to the idea of a body existing through two frameworks ultimately connected by cognitive force:

- **The ‘body’ of a FPDM** = The focal point of multi-dimensional space that connects the inner string + outer string regions + localized EMR consciousness that radiates
- **The body of an animal model such as a human** = The spine and peripheral neural tissue that connects the thoracic + abdominopelvic regions + head where a brain is situated

Important to empirically testing aspects of the theory is the fact that in the phenomenal world, all living organisms with DNA emit electromagnetic radiation. Further, the surface of the bodies of living organisms models with DNA limits the intensity of the radiation. They emit a fraction of the possible radiation at a given wavelength, and this value is called emissivity. It ranges between 0 and 1 (i.e., the theoretical value of a black body the theory correlates to FPDMs). Experimental measurements of biological tissues in the 9–11 m range have shown that absorptivity and emissivity are between 0.9 and 0.97 regardless of the observed color of the surface [11]. Ongoing research uses infrared thermal imaging (IRT) to test aspects of the FoK-FIP theory. The reactions of the sensitive cognitive force to the interoception of FoK-FIP is the mechanism modulating the cognitive broadcasting of signals occurring through the localized EMR consciousness wavefront. Through higher-end cognitive broadcasting, the cognitive force’s reactions correlate with the reactions of the study’s participants, which are dogs with anxiety conditions.

### Figures thematically composed to promote clarity and illustrate causal processes outlined by the FoK-FIP theory (see Appendix)

- Figure A1
- Figure A2
- Figure A3
- Figure A4
- Figure A5
- Figure A6
- Figure A7
- Figure A8

## The rationale underlying the transdisciplinary modeling related to the FoK-FIP system’s core components and comments about how the theory contributes uniquely to the literature

- **For the purpose of internal consistency**, the cognitive force is the fifth fundamental force. This means that the theory’s transdisciplinary modeling includes that the four physical forces of electromagnetic, strong, weak, and gravitational occur through the attributes of the mind interacting ultimately. This concept also applies to the nonphysical force, the cognitive force. The concept here is that only when FPDMs with the pure mental state and normal matter with the state of mental images interact through cosmological frameworks (situated on the universe’s substrate of the attribute dark energy with the pure awareness state) will the cognitive force emerge within the framework. The theory includes five fundamental forces for internal consistency related to this transdisciplinary modeling.
- **The FoK-FIP theory contributes to string theory uniquely** and does this through a paradigm shift. The underlying assumption is that string theory is limited to mathematical theorizing. The FoK-FIP theory that includes aspects of string theory shows a fundamental change in how string theory is approached, which is logical. A significant problem with string theory is that it has little relevance to addressing the issues of ‘life.’ This concept correlates to a statement attributed to physicist Stephen Hawking [33]): “When we understand string theory, we will know how the universe began. It won’t have much effect on how we live, but it is important to understand where we come from and what we can expect to find as we explore.” This could be regarded as the cornerstone of research in the field of string theory and related subfields. Still, a core problem with string theory is its relevance to the phenomenal world. This means that string theory must be ‘beautifully’ described using mathematical language but does not know where it is going. This “any road will work as long as it can be described mathematically” approach has arguably been the road that has led string theory to irrelevance. The transdisciplinary modeling of the FoK-FIP theory gives strings’ vibrations a role through FPDMs related to the framework within a framework approach. This role is defined through text and figures. The aspects of the FoK-FIP theory that include string vibration are based on logic expressed through transdisciplinary modeling. This logic can be extrapolated to include mathematical language with a new criterion. Instead of mathematical language being used to elaborate ‘beautifully’ but irrelevant formulas only accessible to a few that have become very specialized, there is a new criterion. The mathematical language of string theory must contribute to transdisciplinary understanding; specifically, the language must be used to show the relationship between the frameworks (i.e., the cellular framework that occurs through the cosmological). In this process, the mathematical language of string theory contributes to the aim of the FoK-FIP theory, which is to facilitate transdisciplinary modeling that bridges scientific disciplines by facilitating a new understanding of how the universe functions. This sets a new course for string theory where mathematical language is ‘kept in check.’

Further, the FoK-FIP theory represents a new approach to string theory that has led to empirical research, as previously mentioned through studies using living organism models with DNA. The cellular framework within a cosmological framework approach of the theory represents something introduced into the scientific literature that has not been done previously. This approach paves the way to address one of string theory’s biggest problems: Its need for more contact with experiments [34]. According to the FoK-FIP theory, interaction contributes significantly to the phenomenal world’s problems. The theory includes aspects of string theory (i.e., a unified theory of all interactions) through modeling the configuration of FPDMs. The cognitive force emerges through FPDMs with sensitivity to the interoception of FoK-FIP, in which this sensitivity can be abnormal, affecting cognitive broadcasting. This includes higher-end cognitive broadcasting that produces the phenomenal world where the living organism models representing the cellular frameworks emerge. FoK-FIP D is the maladaptive schema of the theory connecting disease to the cognitive force. In contrast, the FoK-FIP MBPI is the treatment for FoK-FIP D introduced into the literature. As such, transdisciplinary modeling is how the FoK-FIP theory contributes to string theory uniquely.

- **Is it possible to convert energy to dark matter through strings?** Theoretically, yes, as was shown when the FoK-FIP theory introduced this idea into the peer-reviewed scientific literature in 2020. Further, the way dark matter is modeled in the theory, although referred to as matter, is unlike normal matter and is not like dark energy. Instead, each FPDM is derived from dark energy and consists of a focal point of dynamical energy or a field with an inner and outer layer of vibrating dark energy strings. Further, dark matter and energy are two fundamental concepts of which little is known. As such, who is qualified to say that the way the FoK-FIP theory models the universe is wrong? In the theory, gravity is a part of the interactions between dark energy, FPDMs, and normal matter, and this aligns with what is known (i.e., matter and energy are the sources of gravitation). The theory converts dark matter differently than normal matter. It does so with dark matter as the reason for the gravitational force, which is in line with the belief of some astrophysicists. The important question is whether the theory’s transdisciplinary modeling is theoretically plausible, and the answer is also yes. It is plausible that the unknown hypothetical energy (i.e., dark energy) responsible for the universe’s accelerated expansion could exist relative to the hypothetical loosely interacting particles that hypothetically comprise a large portion of the universe’s mass (i.e., dark matter and normal matter). Arguably, a theory’s transdisciplinary modeling is plausible if it leads to an approved study design carried out in the testing environment that produces empirical data. The FoK-FIP theory has satisfied these requirements in which the framework within a framework approach is integral to how it has done that. Further, the theory’s transdisciplinary modeling is expected to be used in future research that expands its scope of use.

The theory’s transdisciplinary modeling represents a broadly reaching process where logic guides the course. This process refers to thought experiments where a range of concepts from all the major branches of science are correlated. The theory’s explanation for general observations, which includes why and how they occur, is always internally consistent. Perception is the domain of the cognitive force that cognitively broadcasts signals after emerging within cosmological frameworks. Cognitive broadcasting is modeled as a dynamic process that produces varying degrees of inferential reasoning related to the viewpoint of the cognitive force of cosmological frameworks. The theory includes the idea that cognitive broadcasting is a process understood to be connected to perception. It does not try to reify what emerges within the phenomenal world of interoceptive cognition consciousness. Neither does the theory take a nihilistic approach by saying that phenomena perceived by the cognitive force are not ‘real.’ Instead, the theory acknowledges that the cognitive force through cognitive broadcasting creates a viewpoint and has more or less an understanding of this process that creates what it perceives. The schemas in the scientific literature are expected to be continuously upgraded, as this has been the trend throughout history. The transdisciplinary modeling of the FoK-FIP theory, having explained that literature occurs through cognitive broadcasting by the cognitive force, is not affected by scientific trends. This means that rather than the entire theoretical framework collapsing based on what emerges within the scientific literature, the theory responds by making changes related to the hypothesis based on empirical research findings if needed.

## Discussion

**Figure A4.**
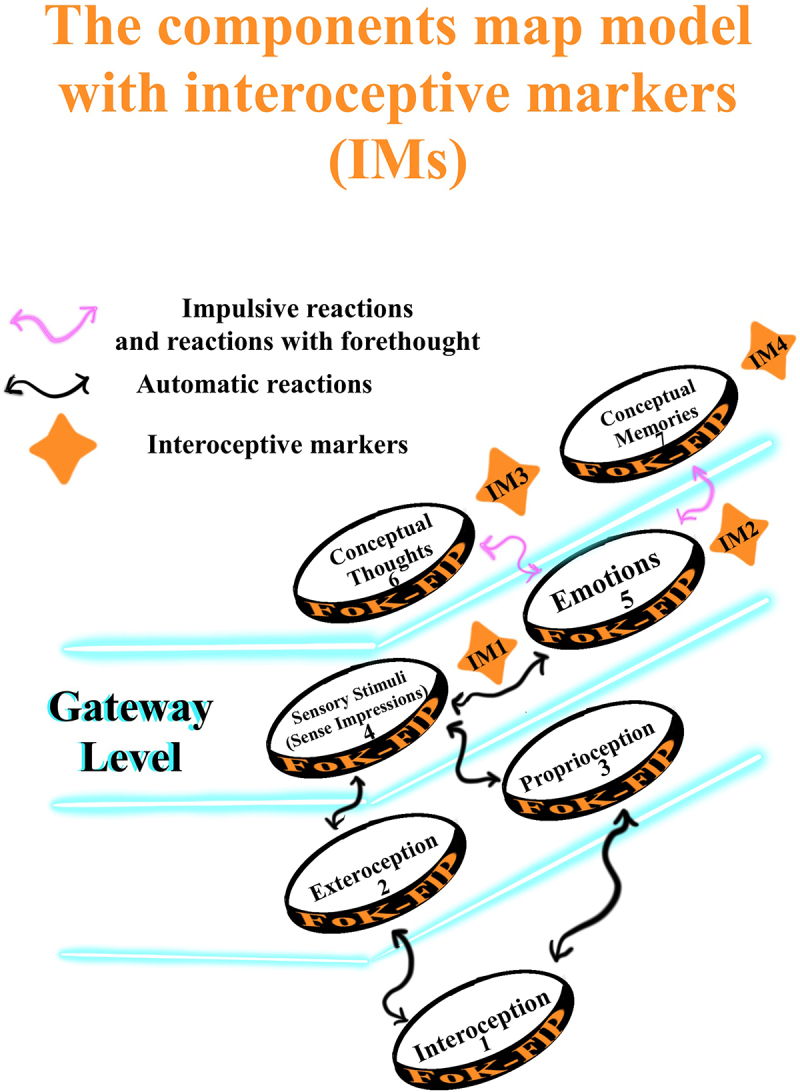
The components map model with interoceptive markers: the components map model depicted in this figure shows where broadcasting signals cognitively by the cognitive force includes emotional processing. As such, an animal model would be the electromagnetic entity representing the cellular framework.

The components map model with IMs (see Appendix Figure A4) is how to envision cognitive broadcasting by the cognitive force. Further, cognitive broadcasting patterns are not static and can be improved and updated with new information related to signals derived from the wavefront. FoK-FIP interference is how the cognitive force learns new information without awareness. Previous cognitive broadcasting patterns are stored through the ELF magnetic field FIP. The initial stages of cognitive broadcasting include the cognitive force reacting to FoK-FIP interference without awareness. Reactions at this stage of the cognitive broadcasting process are automatic, and adaption to signals takes place within a stable context to create interoception, exteroception, and proprioception (i.e., components 1, 2, and 3). In contrast, the gateway level is the intermediate stage of the process that includes the conversion of the sensory stimuli FoK-FIP interference to FoK-FIP feeling tones (e.g., pleasant, unpleasant, or neutral) through automatic reactions by the cognitive force. In this process, awareness of the cognitive force emerges in steps that begin with a ‘sense of signals’ termed sense impressions (i.e., component 4). Further, through the melding of processes below the gateway by the cognitive force that reacts automatically, aspects of the gateway level of cognitive broadcasting include sensorimotor perception. Importantly, the signals cognitively broadcast by the cognitive force to create sensations and the impression of them (i.e., sense impressions) may or may not lead to emotional processing (e.g., happiness, sadness, fear, anger, surprise, embarrassment, jealousy, guilt, and pride) [35]. Cognitive broadcasting for emotions is based on the drive created through the cognitive force’s automatic reactions. In this context, emotional processing occurs through automatic reactions that begin without awareness [36,37] and convert to awareness, in which impulsive reactions follow emotional processing. The core concept related to the gateway level is that the cognitive force develops the ‘sense of self’ that connects to processes above the gateway level that begin through impulsive reactions. In the FoK-FIP theory, perceptual integration precedes conscious access. This concept correlates to cognitive broadcasting processes above the gateway level. Above the gateway level, the cognitive force broadcasts signals to create higher-end cognitive broadcasting correlated to conceptual thoughts (i.e., component 6) and conceptual memories (component 7). The term conceptual refers to aspects of the phenomenal world of interoceptive cognition consciousness (i.e., ‘life’). The cognitive force perceives these aspects as ‘objects,’ including electrons, hadrons, atoms, molecules, mathematical formulas, and living things (i.e., electromagnetic entities) [23].

### FoK-FIP feeling tones will or will not lead to emotional processing

The theory’s transdisciplinary modeling includes the concept that plant models represent cognitive broadcasting devoid of emotional processing, whereas animal models include emotional processing. The core idea here is that emotional processing causes the cognitive force to broadcast for higher-end cognition in a certain way. Related to emotional processing is the idea that the cognitive force that automatically and impulsively reacts to FoK-FIP feeling tones causes ‘attachment’ and ‘aversion.’ This modeling is important because the cognitive force’s ‘attachment’ and ‘aversion’ do not occur through higher-end cognitive broadcasting. This means that they are not based on the perception by the cognitive force of electromagnetic entities or objects (e.g., people, places, and things). Instead ‘attachment’ and ‘aversion’ begin as mid-level cognitive broadcasting (i.e., gateway level) that precedes higher-end cognitive broadcasting (i.e., above the gateway level) and are intimately connected with FoK-FIP feeling tones. Further, feeling tones are integral to the theory’s development of emotional biases with an expanded definition. Emotional biases in the FoK-FIP theory refer to the process where the cognitive force is spontaneously aware of gateway-level processing that occurs through its automatic and impulsive reactions to FoK-FIP feeling tones (e.g., pleasant, unpleasant, or neutral). In this process, the cognitive force makes decisions about how it broadcasts signals cognitively to create deeply rooted personal experiences that will link to higher-end cognitive broadcasting (i.e., above the gateway level). A commonly held assumption may be that one desires or is repulsed by the phenomenon in the world of interoceptive cognition consciousness. Integral to the FoK-FIP theory’s transdisciplinary modeling is the idea that ‘attachment’ and ‘aversion’ arise in reaction to the FoK-FIP feeling tone itself rather than higher-end cognitive broadcasting. The theory’s transdisciplinary modeling of these concepts aligns with the Buddhist psychological model and the concept of feeling tones (see [38]). Related concepts are a *cognitive match* and *cognitive error* that can occur through higher-end cognitive broadcasting. These terms in the FoK-FIP theory refer to instances where the cognitive force associates a very particular interoceptive experience gateway level created through FoK-FIP feeling tones to aspects of the phenomenal world of interoceptive cognition consciousness (i.e., ’life’) above the gateway level:

- **With a cognitive match**, the feeling will match contextual aspects of the phenomenal world. For example, the interoceptive experience created through pleasant FoK-FIP feeling tones at the gateway level links with animal models displaying acts of kindness.
- **With a cognitive mismatch**, the feeling will not match contextually with what emerges through interoceptive cognition consciousness. For example, the interoceptive experience created through pleasant FoK-FIP feeling tones at the gateway level links with animal models displaying acts of violence.

The idea that all conscious percepts are associated with either attachment or aversion is well-trodden ground, especially in Buddhist psychology, as is the distinction between reacting versus responding [39]. However, these concepts are expanded through the theory’s transdisciplinary modeling and framework within a framework approach that has led to empirical research. Through the theory’s transdisciplinary modeling of FoK-FIP D, quantum mechanics implications and the cognitive force connect to cellular frameworks. In cellular frameworks represented by animal models, FoK-FIP D occurs through FoK gene expression with abnormal sensitivity of the cognitive force linked to genetic and/or epigenetic factors (i.e., some external factor influences gene expression). In this process, aspects of sympathetic hyperactivity occur that infrared thermal imaging (IRT) can capture. There are three variations of FoK-FIP D:

1. FoK gene expression is increased
2. FoK gene expression is decreased
3. FoK gene mutation occurs.

### ‘Distress’ = FoK + FIP

The theory’s transdisciplinary modeling for cognitive broadcasting includes the idea that FoK refers to the ‘fear expression’ of the cognitive force, whereas ‘anxiety learning’ occurs through ELF magnetic field FIP patterns. Higher-end cognitive broadcasting is how the cognitive force’s ‘fear’ and ‘anxiety’ manifest in complex ways related to living organism models with DNA. For example, the behavioral responses of animal models are associated with vigilance or hypervigilance, obsessions, or abnormal obsessions. The interoception of FoK-FIP is the construct that correlates to the operational definition of ‘fear’ of the cognitive force. ‘fear’ of the cognitive force is an intervening variable between the FoK awareness charge and awareness current. These ideas align with researcher Ralph Adolph’s Biology of Fear theory [40]. FoK-FIP interference and FoK-FIP feeling tones are the sets of context-dependent stimuli intensity and frequency leading to the cognitive force’s behavioral responses through automatic, impulsive, and forethought reactions. Further, the ‘anxiety’ of the cognitive force is the variable that follows FoK through FIP.’ It is intimately connected to feeling the heat and the patterns of the ELF magnetic fields associated with FoK-FIP feeling tones. FIP is how impulse is experienced by the cognitive force that pushes and pulls in ways that meld with cognitive broadcasting for memory.

### The sensitivity of the cognitive force to the interoception of FoK-FIP

The sensitivity of the cognitive force is the core regulatory concept of the FoK-FIP theory. It explains the cognitive force’s reactions (automatic, impulsive, and reactions with forethought). Sensitivity in this context represents state-dependent dynamic rules that correlate to unawareness of FoK-FIP interference and awareness of FoK-FIP feeling tones (e.g., pleasant, unpleasant, or neutral). These rules continuously update based on the sensitivity of the cognitive force in which cognitive broadcasting patterns aim toward an equilibrium. The cognitive force’s intrinsic (derived from FoK) and extrinsic (derived from FIP) polarities are the mechanistic explanation for ‘distress.’ These intrinsic and extrinsic properties refer to the cognitive force being ‘bipolar’ with an expanded definition.

- **The ‘bipolar’ nature of the cognitive force** = intrinsic polarity from FoK (that leads to the awareness current) + extrinsic polarity from FIP.

### Indeterminism or uncertainty of cognitive broadcasting that connects to motivation

In the FoK-FIP theory, uncertainty is related to the interoception of FoK-FIP. Indeterminism or uncertainty comes through cognitive broadcasting and is ultimately related to the reactions of the cognitive force based on its sensitivity to either FoK-FIP interference or FoK-FIP feeling tones (e.g., pleasant, unpleasant, or neutral). This sensitivity makes the cognitive force susceptible to being driven to cognitively broadcast signals in a certain way, denoted by the component map model’s interoceptive markers (IM1–IM4). The sensitivity of the cognitive force explains how FoK and FIP interfere with each other ultimately. Further, FoK and FIP are set pairs of quantities in which the more precisely the cognitive force knows one, the less precisely it knows the other. In this process, the cognitive force emerges from FoK and is pushed by FIP and thus comes between them but cannot prevent or alter this course of events. Instead, the cognitive force with degrees of sensitivity responds to the quantitative value of the interoception of FoK-FIP. The cognitive force reacts in this process, allowing it to bind to parts of the wavefront that, without doing that, it could not cognitively broadcast, which it will do with degrees of primordial motivation.

- **Primordial motivation** = The cognitive force with ‘distress’ reacts automatically to FoK-FIP feeling tones at the gateway + emotional processing + impulsive reactions + higher-end cognitive broadcasting that displays animal models engaged in activities associated with fundamental needs.

The electromagnetic entities of animal models driven primordially are displayed in the phenomenal world of interoceptive cognition consciousness. This means that their behaviors revolve around three fundamental categories of need:

- Food
- Fluid
- Sex

### Relative statements about the perception of the phenomenal world of interoceptive cognition consciousness (i.e., ‘life’) by the cognitive force

Relative in this context means related to cognitive broadcasting. In the FoK-FIP theory, the perception of the cognitive force is based on its core responses to the interoception of FoK-FIP.

In this process, signals broadcast cognitively by the cognitive force need to be considered and judged in relation to the FoK and the FIP. Relative frequency of the interoception of FoK-FIP refers to the number of times a particular value of FoK or FIP appears in a data set consisting of signals derived from the electromagnetic field. It indicates how often a specific event of FoK-FIP interference or FoK-FIP feeling tones occurs within the total number of cognitive cycles occurring through the wavefront.

- **Relative frequency of the interoception of FoK-FIP** = The number of times FoK or FIP occurs divided by the total number of FoK-FIP interference or FoK-FIP feeling tone events occurring in a given scenario (e.g., cognitive cycle).
- **FoK-FIP influence** is the nonphysical message of FoK-FIP interference that leads to FoK-FIP feeling tones, resulting in either a change or reinforcement in awareness or acceptance by the cognitive force. These effects can be positive or negative, abrupt or gradual, short-lived or long-lasting. Further, not all effects related to the interoception of FoK-FIP result in change; some signal messages created through reactions to FoK-FIP feeling tones reinforce an existing belief. The point here is that when the cognitive force reacts to FoK-FIP feeling tones, it cognitively broadcasts with awareness to create belief systems, attitudes, and emotional, physiological, and behavioral effects. These effects through higher-end cognition are displayed through the animal models representing the cellular frameworks that occur through cosmological frameworks.

### The steps of cognitive broadcasting that create the ‘I’ of the cognitive force

1. The cognitive force with varying degrees of ‘distress’ reacts to FoK-FIP interference using automatic reactions during lower level (i.e., below the gateway level) cognitive broadcasting. In this process, it is unaware of its existence. The following statement can summarize this process: “I automatically react without needing to ‘see’ myself, nor do I need to be aware that I exist.”
2. The cognitive force with varying degrees of ‘distress’ reacts automatically to FoK-FIP feeling tones during mid-level (i.e., gateway level) cognitive broadcasting. It is aware of its existence, which may include varying degrees of heat linked with patterns of the ELF magnetic field FIP in which emotional processing may follow or not. The following statement can summarize this process: “I automatically react without needing to ‘see’ myself, but I am aware that I exist through a very specific pleasant, unpleasant, or neutral feeling.”
3. If there is emotional processing, then after that cognitive broadcasting, the cognitive force responds to FoK-FIP feeling tone using impulsive reactions only or impulsive reactions followed by reactions with forethought. If this process leads to higher-end cognitive broadcasting (i.e., above the gateway), the cognitive force will perceive aspects of the phenomenal world to varying degrees. Higher-end cognitive broadcasting produces degrees of inferential reasoning linked with a specific pleasant, unpleasant, or neutral feeling. In this process, the cognitive force perceives the animal model (e.g., human or non-human), representing the cellular framework. By perceiving this animal model, the cognitive force will derive a sense of self to varying degrees. However, it can do so with very different beliefs about ‘I,’ specifically if the representative of the cellular framework is human. The viewpoint may be:
  - I am a living organism model with DNA.
  - I am the cognitive force.

### Suffering versus happines*s*

‘Suffering’ and ‘happiness’ refer to the cognitive force that cognitively broadcasts to create emotional processing. These terms specifically refer to how the cognitive force experiences FoK-FIP feeling tones. As such, the theory’s transdisciplinary modeling of these concepts differs from the current literature. Further, these concepts include the terms ‘relative’ and ‘absolute.’ In this context, ‘relative’ is associated with a lack of durability and may only last for a short time, whereas ‘absolute’ is associated with being durable and thus lasting for a long time. The core idea related to the difference between these terms is that “relative happiness” depends on a feeling, whereas “absolute happiness” ultimately depends on self-efficacy. The self-efficacy of the cognitive force in the FoK-FIP theory is dependent on its ability to initiate a reaction with forethought following impulsive reactions. This aligns with social cognitive theory [41], in which self-efficacy is defined as the belief in one’s capabilities to mobilize the motivation, cognitive resources, and courses of action needed to meet given situational demands. The transdisciplinary modeling of the FoK-FIP theory includes the concept of ‘love’ expanded by connecting it to FoK-FIP feeling tones.

- **Suffering** = Cognitive force that perceives FoK-FIP feeling tones as unpleasant then automatically reacts with aversion + cognitive broadcasting with emotional processing followed by impulsive reactions only + higher-end cognition in which animal models represent the cellular frameworks. These animal models are in a state of undergoing pain, distress, or hardship. This may include being harmed or threatened by others who may hate them in fleeting or durable ways because it is based on feeling.
- **Relative Happiness** = Cognitive force that perceives FoK-FIP feeling tones as pleasant automatically reacts with attachment + cognitive broadcasting with emotional processing followed by impulsive reactions only + higher-end cognition in which animal models represent the cellular frameworks. These animal models engage in pleasurable activities that can include others who fleetingly love them because it is based on a feeling.
- **Absolute Happiness** = Cognitive force that perceives FoK-FIP feeling tones as pleasant, unpleasant, or neutral automatically reacts + cognitive broadcasting with emotional processing followed by impulsive reactions followed by reactions with forethought + higher-end cognition in which animal models represent the cellular frameworks. These animal models may be in a state of undergoing pain, distress, or hardship or not. This may include being harmed or threatened by others, or they may be engaged in pleasurable activity with or without others. Regardless, these animal models have a high degree of inferential reasoning and self-efficacy and love a broad range of others durably because it is not based on a feeling or dependent on the love being reciprocated.

### The mind’s game: interoceptive cognition consciousness

The transdisciplinary modeling of the FoK-FIP theory may help with the problem, as stated by physicist Lee Smolin, that imagination is not productive when asking the wrong question [34]. The big questions the FoK-FIP theory answers are as follows:

- **What are we?** We are the cognitive force.
- **What is ‘life’?** Interoceptive cognition consciousness
- **Why are we here?** Our role in the universe is to cognitively broadcast signals derived from the other attributes of the mind interacting through cosmological frameworks. By doing that, the mind can benefit itself cognitively. In this regard, the role of the cognitive force can be summarized by the following statement based on transdisciplinary modeling: It is logical to benefit others because there are no others ultimately. Instead, there is the mind that, having expressed unlimited potential, manifested a universe that is simply a term for where its attributes interact.

The theory’s transdisciplinary modeling includes the idea that the cognitive force is on a journey as the cognitive broadcaster of interactions between the attributes of the mind. Based on its sensitivity to the interoception of FoK-FIP, its role is to create a science story. The cognitive force is the hero of its modest narrative that unfolds in a way that may be broadly compared to the *hero’s journey* [42]. The storyline includes inner and outer obstacles correlated with the inner and outer layers of strings of FPDMs that ultimately shape the cognitive force’s sensitive viewpoint. The cognitive broadcasting journey of the cognitive force refers to the mind’s game interoceptive cognition consciousness. The FPDMs are the scorekeepers through strings that convert, whereas the cognitive force is the perceiver of the game that plays out as a mental movie through higher-end cognitive broadcasting. The complexity of the mind’s game includes the idea that higher-end cognitive broadcasting creates illusions. This means that aspects of the three-dimensional phenomenal world of interoceptive cognition consciousness emerge in ways that make them appear solid. Nevertheless, phenomena do not solidly exist as they seem to. In this regard, interoceptive cognition consciousness represents a complex mind game; we are the game’s players.

The cognitive force can perceive how it is doing in the game through the animal models as representatives of the cellular frameworks. How animal models (e.g., human and non-human) interact with others correlates with the reactions of the cognitive force to FoK-FIP feeling tones. The volitional reactions (e.g., impulsive reaction only or impulsive reaction followed by reaction with forethought) of the cognitive force result in string conversion. FPDMs with more open strings than closed strings correlate with the viewpoint of the cognitive force with less inferential reasoning. In contrast, FPDMs with more closed strings than open strings correlate with the viewpoint of the cognitive force with more inferential reasoning. In this process, the cognitive force creates an understanding of its existence. The theory’s transdisciplinary modeling of strings’ conversion includes the idea that the motivation of the cognitive force in using impulsive reactions alone or impulsive reactions with forethought to broadcast for higher-end cognition will result in more or less conversion of strings. This idea aligns with karma originating in the Rig Veda of Hindu literature. In Sanskrit, it means ‘action’ that the FoK-FIP theory correlates with reactions of the cognitive force, specifically the volitional reactions.

Localized EMR consciousness distorts when it is pulled into the configuration of FPDMs at the end of a cognitive broadcasting cycle. In this process, information associated with the wavefront is distorted, amounting to a data transfer through a purely mental process of FPDMs. The concept here is that when the conical EMR distorts, and through a purely mental process, FPDMs recognize the cognitive force’s impulsive reactions or reactions with forethought and ‘tallies’ the results through string’ conversion. In this tallying process, the pure mental state of FPDMs controls conversion (i.e., how many open and closed strings convert). Further, in the universe between each FPDM, there is a unique relationship to cognitive force, creating a certain continuity related to cognitive broadcasting through the mind’s attributes interacting. Within cosmological frameworks, the cognitive force will react to the interoception of FoK-FIP, and FPDMs will learn its responses through strings that convert. In this way, the cognitive force’s responses directly affect its relationship to FPDMs through strings’ conversion. This feedback loop has short-term effects on how the FPDMs process their simultaneous relationship with normal matter and dark energy through cosmological frameworks.

This feedback loop has long-term cyclic, although not repetitive, effects related to the cognitive broadcasting journey of the cognitive force through its relationship to FPDMs. Each FPDM will cause the emergence of the same cognitive force through many cosmological frameworks to create the journey. Although this journey plays out through higher-end cognitive broadcasting, the cognitive force is always behind the scenes as the invisible observer of interoceptive cognition consciousness. Further, in this journey, many ‘birth’ and ‘death’ events mark the beginning and end of game rounds that correlate with animal models’ birth and death in the phenomenal world. This concept aligns with the Tibetan Buddhist concept of the kinds of *bardo*, which includes where the term refers to an intermediate state, the period between death and rebirth [19].

### The ‘status quo’ or the ‘big transformation’

The theory’s transdisciplinary modeling includes the idea that complexity refers to the processing of signals by the cognitive force generated through the cosmological frameworks. Aspects of the sensitivity of the cognitive force, while cognitive broadcasting emerge into the phenomenal world of interoceptive cognition consciousness through the networking of animal models (e.g., human and non-human). This means working or not working together to create the complexity of ‘life’s’ relationships. A core idea is that through higher-end cognitive broadcasting, the viewpoint of the cognitive force includes *’ego’* to varying degrees. In this context, this term refers to a sense of self-esteem or self-importance the cognitive force derives from animal models (e.g., human or non-human) representing cellular frameworks. Human models are important in ways associated with the universe’s layout, developed through the theory’s schema of the mind’s game (i.e., interoceptive cognition consciousness). The importance of human models is that they indicate how the cognitive force can reach a level of complexity related to cognitive broadcasting based on sensitivity to the interoception of FoK-FIP that can positively or negatively affect broadly other cosmological frameworks. This concept correlates with two game scenarios: the ‘Status Quo’ or the ‘Big Transformation.’

The ‘Status Quo’ in the FoK-FIP theory means the existing state of affairs related to cognitive broadcasting by the cognitive force. In this context, the cognitive force with abnormal sensitivity to the interoception of FoK-FIP is “driven out of control” by FPDMs. The core idea here is that this accurately describes the current phenomenal world situation and correlates with the rise in FoK-FIP D. In contrast, the ‘Big Transformation’ is where the cognitive force tames FPDMs ultimately through reactions with forethought and correlates with the FoK-FIP MBPI. In this process, when reactions with forethought consistently follow impulsive reactions, it causes the conversion of the strings of FPDMs, allowing the cognitive force to perceive the interoception of FoK-FIP differently, specifically through a changed sensitivity. The cognitive force can broadly alleviate its ‘suffering’ if this happens through many cosmological FoK-FIP frameworks. Although this has not occurred, it could potentially happen. This scenario includes the cognitive force escaping from the ‘black hole of relationship’ with FPDMs. Escape is synonymous with the mind benefitting itself through the cognitive force that consistently benefits others in ways not based on feelings. Further, transdisciplinary modeling includes the notion that the population of strings of FPDMs must be predominantly closed for the cognitive force to win the game of interoceptive cognition consciousness. In this process, FPDMs convert back to dark energy, where strings disappear into the pure awareness state. This concept has aspects that align with the quantum theory, enabling energy information to escape. Importantly, the mind itself does not change when the ‘Status Quo’ or the ‘Big Transformation’ happens.

## Conclusion

This paper has introduced the FoK-FIP system to show how the FoK-FIP theory contributes broadly to the scientific literature, including theoretical and classical theory, empirical research, biology, medical diagnosis, and intervention.

## Figures and Tables

**Figure A1.**
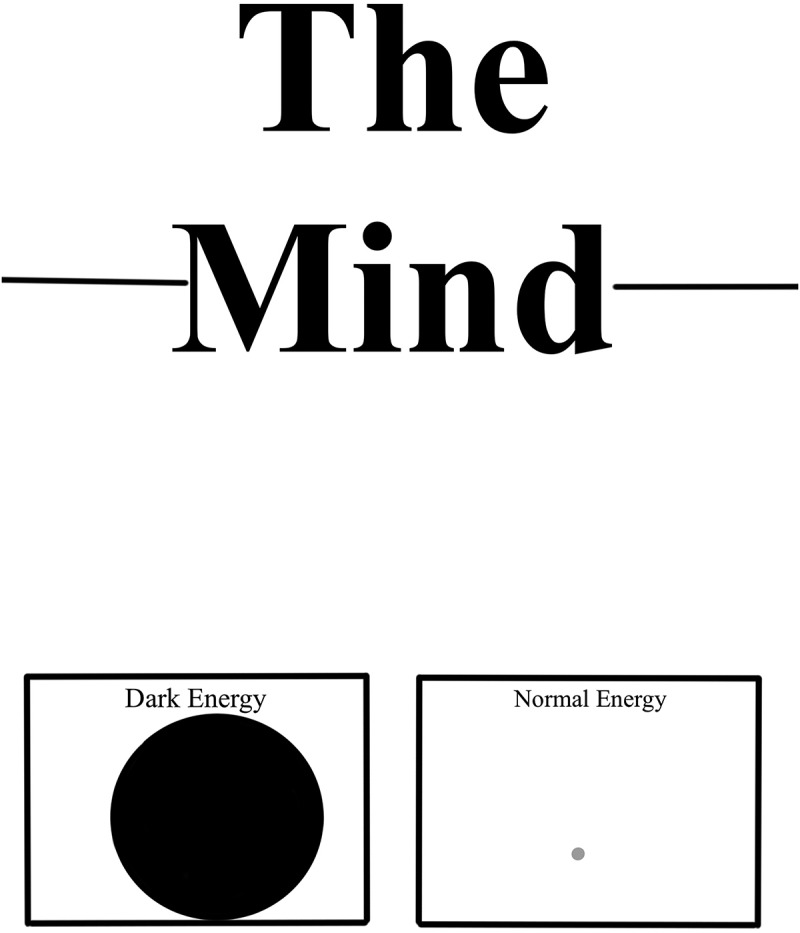
Through the mind’s potential manifesting, more dark energy emerged from this event than normal energy.

**Figure A2.**
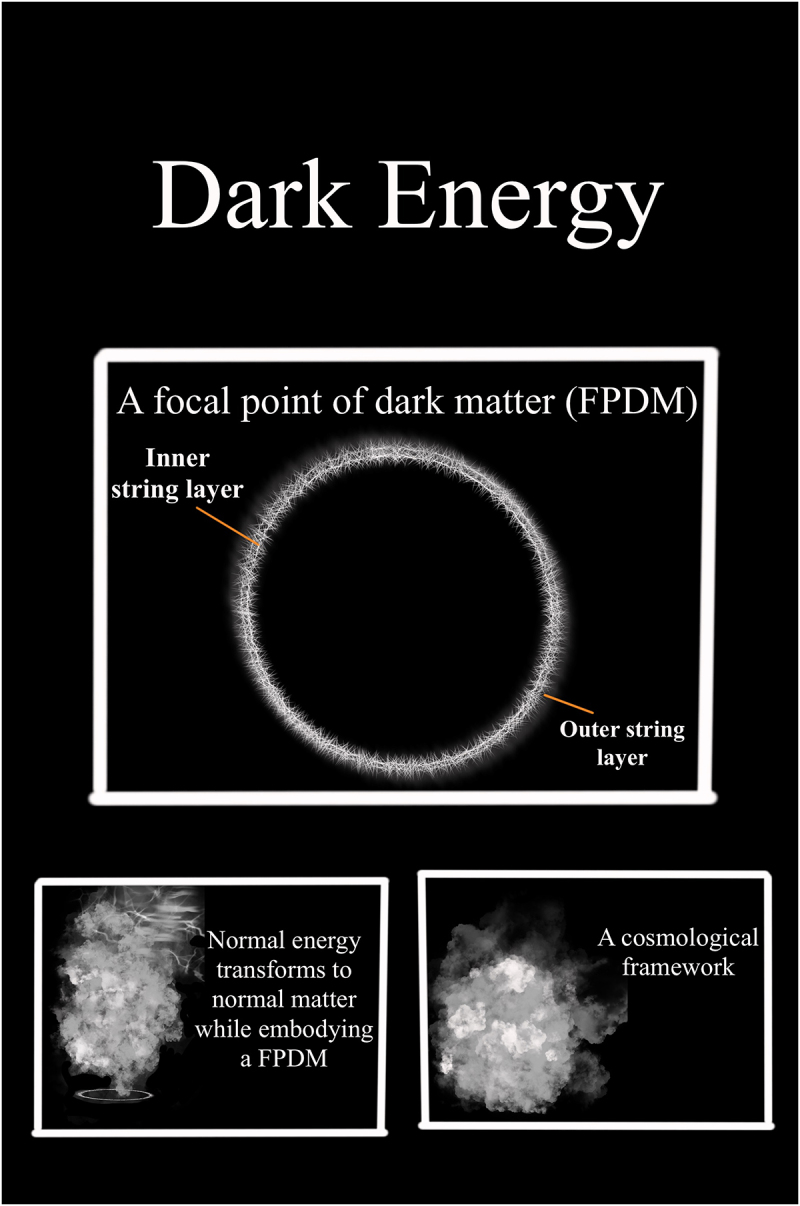
Dark energy transforms to cause the emergence of focal points of dark matter (FPDMs): the configuration of a single focal point of dark matter (FPDM) consists of a focal point of multi-dimensional space surrounded by an inner and outer string layer (Top). Through this transformation, the formation of cosmological frameworks occurs. This figure depicts only one cosmological framework being formed (Bottom).

**Figure A3.**
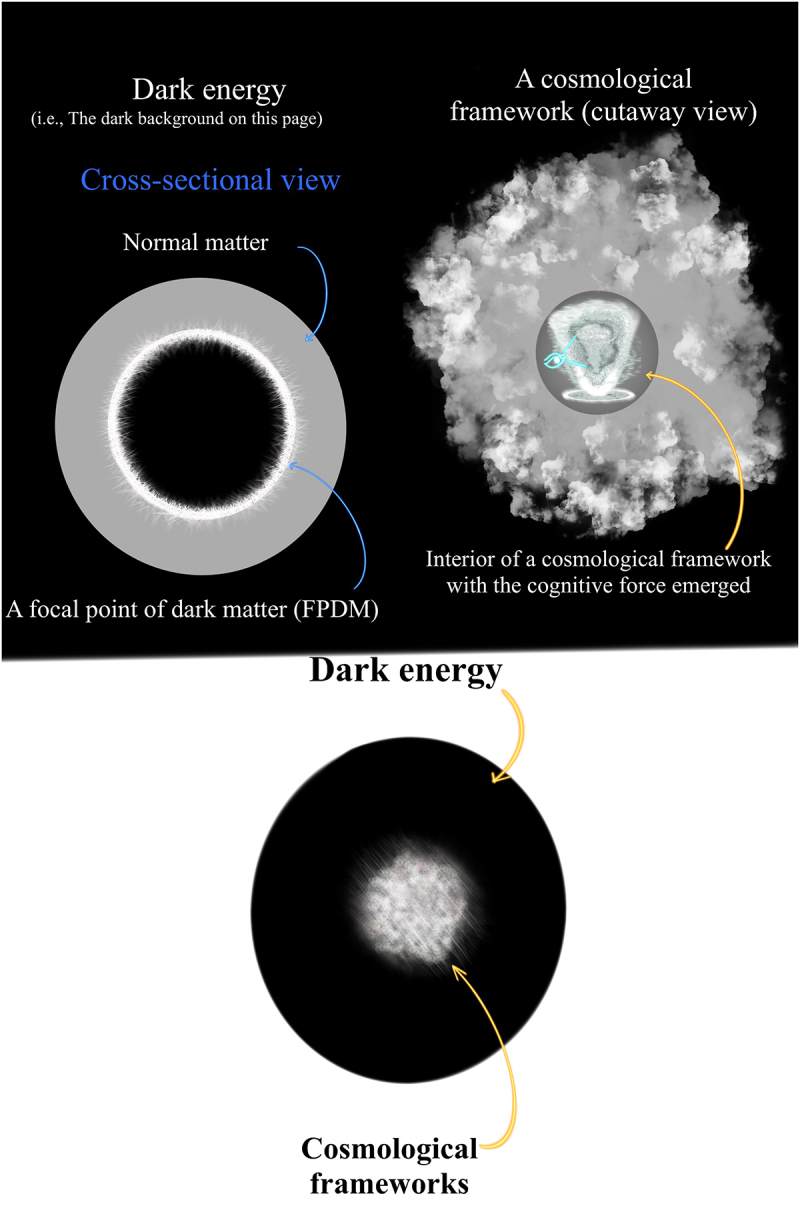
A cosmological framework: these figures show different viewpoints of a cosmological framework through which the cognitive force emerges (Top). The universe is comprised of cosmological frameworks with a dark energy substrate (Bottom).

**Figure A5.**
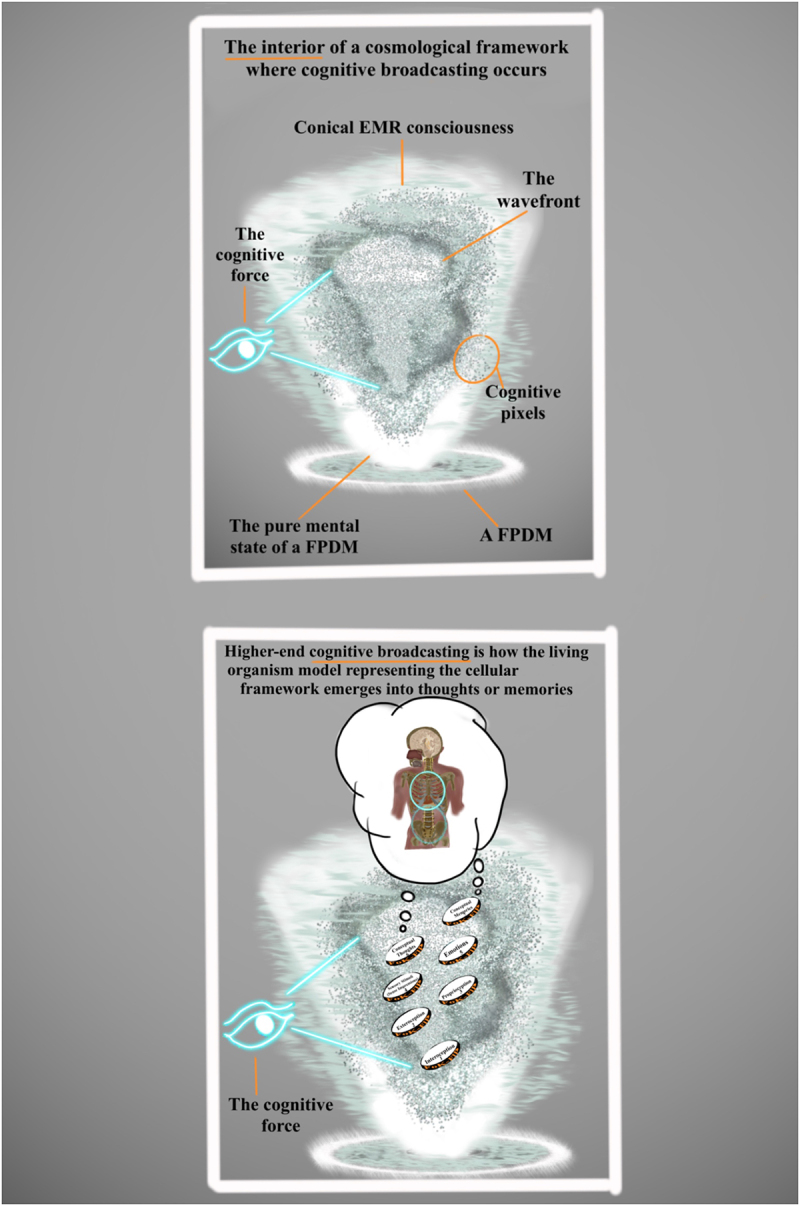
Cognitive broadcasting. The aspects of cognitive broadcasting (Top). The levels of cognitive broadcasting and the components these levels create through the cognitive force broadcasting signals cognitively (Bottom).

**Figure A6.**
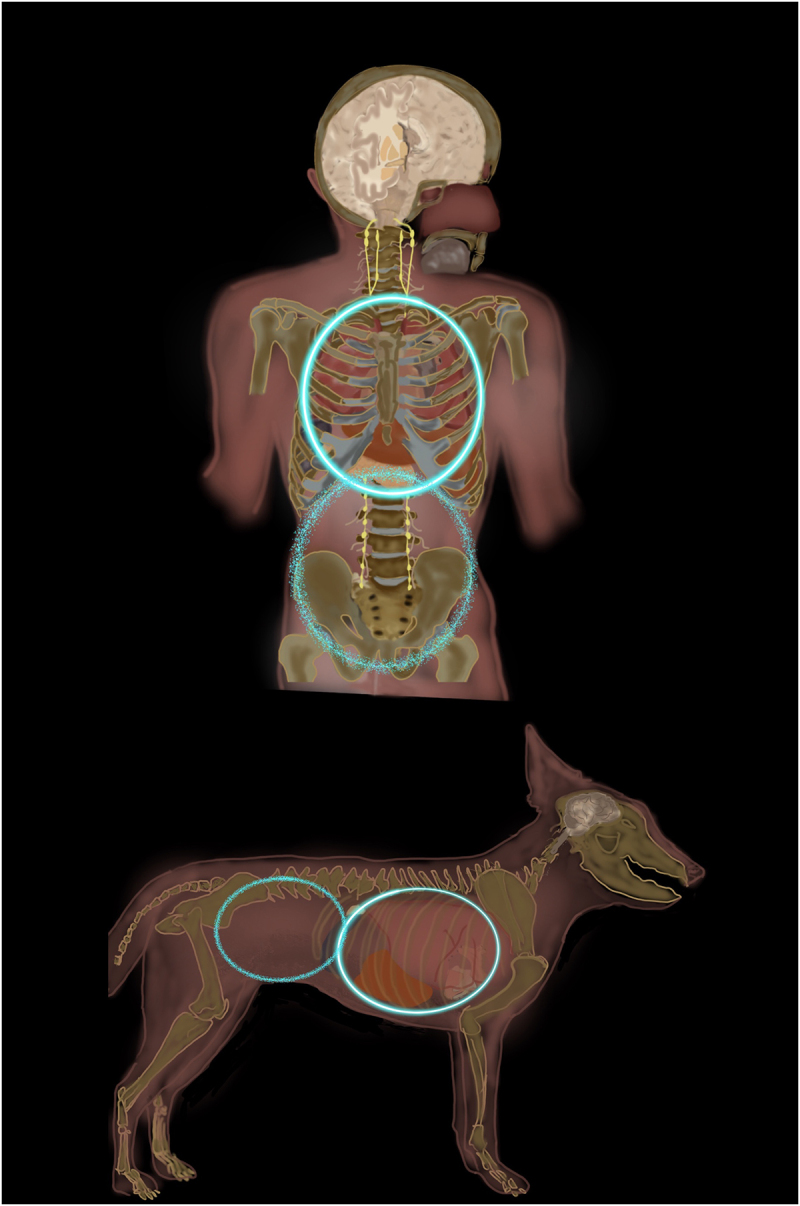
The body of an animal model such as a human and a dog = the spine and peripheral neural tissue that connects the thoracic (demarcated by a solid circle)

^+^abdominopelvic regions (demarcated by a dotted circle) + head where a brain is situated

**Figure A7.**
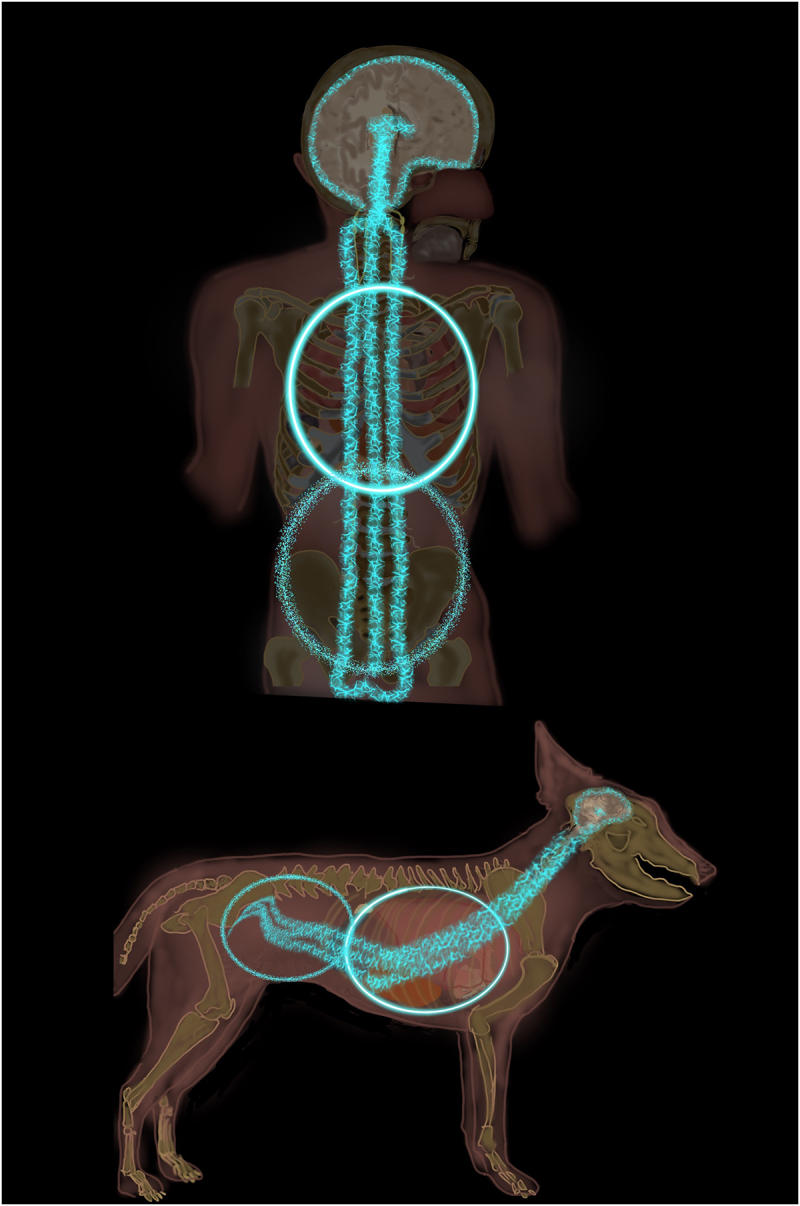
In the cellular frameworks, the cognitive force is part of the coupling between the nonphysical and physical electromagnetic fields associated with living organism models with DNA (representing the cellular framework). For example, this coupling is associated in animal models such as humans (Top) or dogs (Bottom) with the spinal cord and peripheral nervous tissue connecting the torso and head. This concept correlates with magnetic fields of the human body research (see [43]).

**Figure A8.**
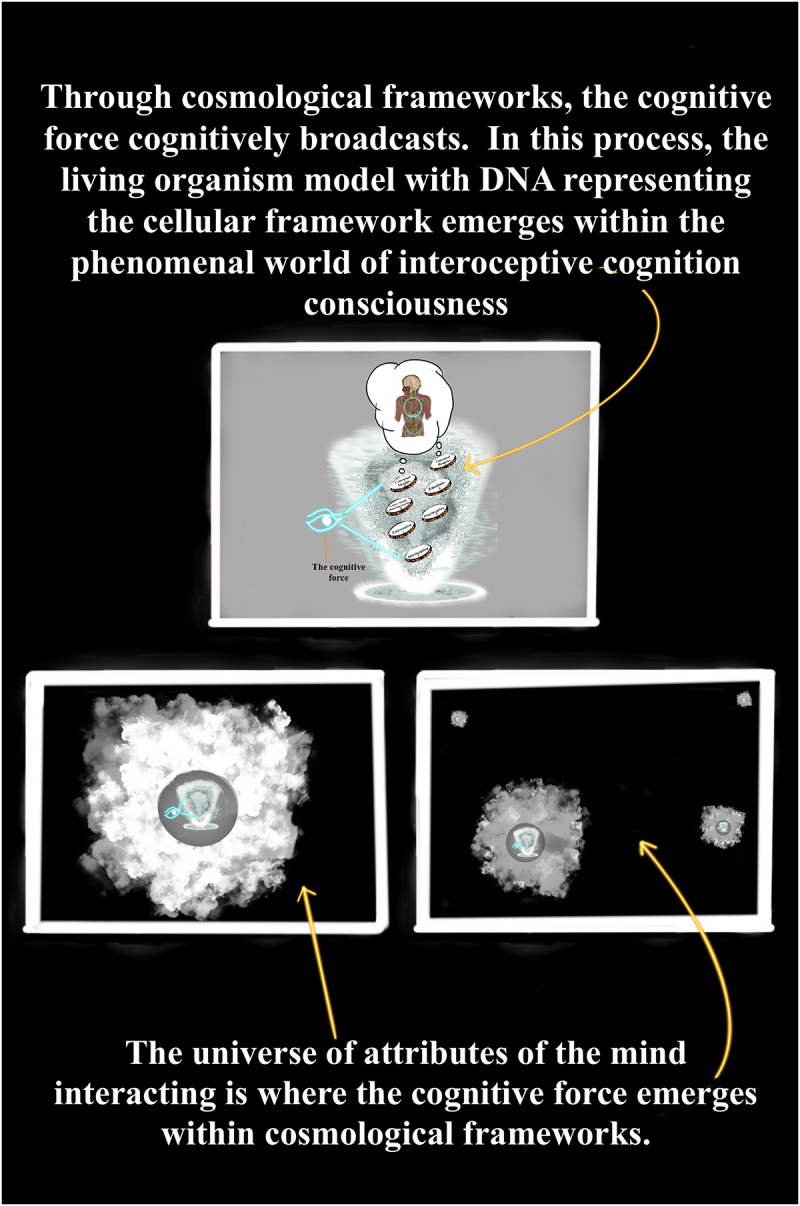
The framework within a framework approach of the FoK-FIP theory.

## Data Availability

There is no data from the study participants to report at this time. The original contributions presented in the study are included in the manuscript; further inquiries can be directed to the corresponding author.
